# Prevalence and changes in food-related hardships by socioeconomic and demographic groups during the COVID-19 pandemic in the UK: A longitudinal panel study

**DOI:** 10.1016/j.lanepe.2021.100125

**Published:** 2021-05-18

**Authors:** Jonathan Koltai, Veronica Toffolutti, Martin McKee, David Stuckler

**Affiliations:** a Sociology Department, University of New Hampshire, Durham, USA; b Centre for Health Economics & Policy Innovation and Department of Economics & Public Policy, Imperial College London, London, UK; c Centre for Global Chronic Conditions, London School of Hygiene and Tropical Medicine, London, UK; d Dondena Centre for Research on Social Dynamics and Public Policy and Department of Social and Political Sciences, Bocconi University, Milan, Italy

**Keywords:** Food-related hardships, Food insecurity, COVID-19, Lockdown measures, Job Retention Scheme, Furlough

## Abstract

**Background:**

Food insecurity concerns have featured prominently in the UK response to the COVID-19 pandemic. We assess changes in the prevalence of food-related hardships in the UK population from April to July 2020.

**Method:**

We analysed longitudinal data on food-related hardships for 11,104 respondents from the April-July 2020 waves of the Understanding Society COVID-19 web survey with linked data from the 2017-9 wave of the annual Understanding Society survey. Outcome variables were reports of being hungry but not eating and of being unable to eat healthy and nutritious food in the last week, which were adapted from the Food Insecurity Experience Scale. We used unadjusted estimates to examine changes in population prevalence and logistic regression to assess the association between employment transitions and both outcomes at the individual level.

**Findings:**

The prevalence of reporting an inability to eat healthy or nutritious food rose from 3•2% in April to 16•3% in July 2020. The largest increases in being unable to eat healthy or nutritious food were among Asian respondents, the self-employed, and 35-44-year-olds. The prevalence of being hungry but not eating rose from 3•3% in April to 5•1% in July, with the largest increases observed among unemployed individuals below age 65. Those moving from employment to unemployment had higher odds of being hungry but not eating in the last week relative to furloughed individuals (OR = 2•2; p < 0•05; 95% CI: 1•1 to 4•2) and to the persistently employed (OR = 3•5; p < 0•001; 95% CI: 1•8 to 6•9), adjusting for age, highest qualification in 2017-19, net household income in 2017-19 (equivalized), gender, race/ethnicity, number children at home (aged 0-4, 5-15, and 16-18), cohabitation status, and government office region. Respondents moving from employment to unemployment also had higher odds of reporting an inability to eat healthy and nutritious food relative to furloughed individuals (OR = 1•9; p < 0•05; 95% CI: 1•4 to 3•2) and to the persistently employed (OR = 2•0; p < 0•01; 95% CI: 1•2 to 3•4). No statistically significant differences were found between furloughed individuals and the persistently employed in their probability of reporting either outcome.

**Interpretation:**

Food-related hardships increased substantially in the UK between April and July 2020, largely driven by reports of an inability to eat healthy and nutritious food. The Coronavirus Job Retention Scheme and Self-Employment Income Support Scheme appeared to have conferred some protection, but more could have been done to mitigate the problems we describe in obtaining affordable food.

**Funding:**

DS is funded by the Wellcome Trust investigator award. JK and DS are funded by the European Research Council n. 313590 – HRES. VT is funded by the European Research Council n. 694145- IFAMID.

**Abbreviations**
United Kingdom (UK): Understanding Society The UK Household Longitudinal Study (UKHLS), UN Global Food Insecurity Experience Scale (FIES)

**Research in Context**

**Evidence before this study**

We searched Google Scholar with the terms “COVID-19” and “food insecurity” and “UK”; and “food insecurity” and “UK” and “coronavirus”, published between January 1^st^ and October 31^st^, 2020. One cross-sectional report was identified, which found higher levels of food insecurity in early April 2020 relative to 2018. Importantly the report relied on items used to measure food insecurity that referred to a 12-month time span in 2018 and then a 30-day time span in April 2020, a potential source of bias for examining changes in population prevalence over time.

**Added value of this study**

Here we provide the first longitudinal national probability study that tracks temporal changes in population prevalence of self-reported lack of nutrition and hunger several months following the initial COVID-19-related lockdown measures in the UK. The prevalence of self-reported lack of nutrition rose for all socioeconomic and demographic and groups from April to July 2020, but did so for some more than others. Some of the largest increases were among Asian respondents, the self-employed, respondents aged 35-44, those with two or more children aged 5-15 in the household, and those living in Scotland, London, and the North West of England and Midlands. Across all sociodemographic characterises included in this study in July, the highest overall prevalence of self-reported hunger was observed among unemployed individuals below age 65.

At the individual level, losing employment was associated with a higher odds of reporting a lack of nutrition and being hungry but not eating in the past week compared to those furloughed under the Coronavirus Job Retention Scheme and the persistently employed. Importantly, furloughed individuals did not differ in their probability of entering either outcome relative to the persistently employed.

**Implication of all the available evidence**

This study documents an alarming increase in the prevalence of food-related hardships in the UK during the pandemic, largely driven by the inability to eat healthy and nutritious food. Unfortunately, given our inability to distinguish the reasons for our findings, specifically whether this was due to inability to afford or to gain access to food, we are limited in what we can propose as a policy response. We do, however, see that the Coronavirus Job Retention Scheme and Self-Employment Income Support Scheme appeared to have conferred some protection, but more could have been done to mitigate the problems we describe in obtaining affordable food. Hence, our main recommendation is that, in this and future pandemics, there should be an early assessment of the food supply system, with a particular focus on the ways that groups within society may be affected differently. This should take full account of the diversity within society. Thus, it is plausible that some groups, such as those in isolated rural areas or who are digitally excluded, may have been especially badly affected even though our data would have been insufficiently granular to detect it.

Alt-text: Unlabelled box

## 1 Introduction

Concerns about food supply have featured prominently in the UK's response to the COVID-19 pandemic [1]. In March 2020, the media reported fights breaking out as people attempted to stock up on rapidly diminishing supplies [2]. Supermarket shelves emptied and food producers asked how they could continue to supply them without placing their workers at risk. The immediate panic resolved but soon gave way to concerns about the many families who, until then, had been just about coping. Austerity measures adopted since 2010 had left many living a precarious existence characterised by insecure employment, income, and in some cases food and shelter [3]. Many were dependent on the growing number of foodbanks(4) and, for those who qualified, free school meals for their children. As schools closed, many families found that they had to find additional food for their children, even though many were facing loss of income or employment. In 2019/20 over 1•4 million school children in England, around 15% of the total, received free school meals [5]. While the rules of entitlement vary among the four nations of the UK, those whose families are receiving certain benefits are likely to be eligible. One recent study estimated that half of free school meal eligible children could not access the scheme in April 2020 [6].

Prior research implicates unemployment, falling wages, and social protection measures as strong determinants of increasing food insecurity [7]. Data from Her Majesty's Revenue and Customs (HMRC) and the Office for National Statistics (ONS) reveal that each of these factors has worsened in the context of COVID-19 [8, 9]. Briefly, since the onset of UK lockdown measures introduced on March 24^th^ 2020, the numbers of people claiming unemployment related benefits rose from 1•2 million in March to 2•1 million in April 2020, increasing to 2•7 million in July; by July 31^st^ 2020, 9.6 million employees had been furloughed by 1•16 million employers. From April-June 2020, the total number of hours worked fell to its lowest level since 1994. The number of seasonally adjusted vacancies hit a record low in the UK between April and June 2020.

In an effort to mitigate these economic shocks, the UK government implemented the Coronavirus Job Retention Scheme (JRS) or ‘furlough’ scheme, whereby employers receive 80 per cent of wages (up to £2,500 per month) in the form of grants in order to keep employees on their payroll [7]. Additionally, the Self-Employment Income Support Scheme (SEISS) issues grants to eligible self-employed individuals adversely affected by the pandemic, corresponding to 80% average monthly trading profits, paid out in a single instalment covering 3 months’ worth of profits (up to £7500) [8]. According to the National Audit Office, while at estimated 12•2 million people have benefitted from support from the UK's employment protection schemes, as many as 2•9 million people were not covered due to ineligibility [12].

To date, little is known about the prevalence and patterning of insecure food circumstances following the lockdown measures in the UK, and whether social protection policies have helped buffer COVID-related economic shocks. This is a critical gap, as the government's response has been criticised for allowing millions to fall through the holes in the social safety net [10]. According to the National Audit Office, while at estimated 12•2 million people have benefitted from support from the UK's employment protection schemes, as many as 2•9 million people were not covered due to ineligibility [11].

Here, we draw on longitudinal data from the UK in order to inform this debate. We test whether the prevalence of reporting being unable to eat health and nutritious food, and reporting being hungry but not eating in the past week has changed from April (beginning of the lockdown measures) to July 2020 across key markers of social inequality. We also assess whether UK's employment protection schemes buffered the impact of unemployment transitions on those outcomes at the individual level.

## 2 Methods

### 2.1 Source of data

Data used in this study are derived from the UK Understanding Society study (or UK Household Longitudinal Study, UKHLS), which is the UK's main longitudinal Household Survey, and one of the largest household panel studies in the world [12], [13], [14]. UKHLS includes a panel survey of more than 40,000 households, beginning in 2009, based on a clustered stratified probability sample of UK households, described in detail in previous research [15]. Following the onset of the pandemic, members of households who participated in either of the two most recent Understanding Society survey waves (Waves 8 or 9), who were older than age 16 years, were invited to complete the Understanding Society COVID-19 web survey (COVID-19 web survey). The first wave of the COVID-19 web survey was collected online between 24th and 30th April 2020 [14]. Invitations and reminders were sent via email, text message, or postal letter. 17,452 completed Wave 1 of the COVID-19 web survey either fully or partially, corresponding to a response rate of 41•2%. A total of 13,754 individuals subsequently participated in the July COVID-19 web survey. Sampling strategies and response rates are explained in greater detail within the documentation provided by the Understanding Society team [14]. The survey weights in the COVID-19 web survey extend the weighting strategy used in the UKHLS annual survey, which adjusts for representativeness and attrition. Detailed procedures used to construct survey weights can be found within the in a recent report. [12, 15]. Table A2 in the appendix provides web links to study materials.

**Fig. 1:**
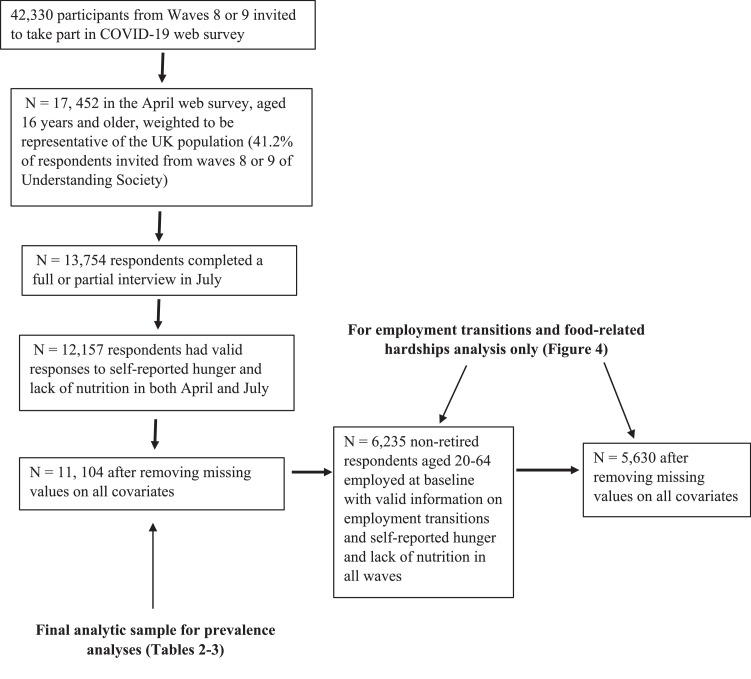
Sample inclusion.

Of the 13,754 participants who completed the July survey, 12,157 respondents had valid responses to both food-related hardship items in both April and July. After removing missing values on all socio-demographic predictors, our analysis of changing food intake perceptions includes 11,104 respondents. Fig. 1 illustrates these sample selection procedures visually.

### 2.2 Measures

Our food-related hardship items are adapted from the Food Insecurity Experience Scale (FIES) [9]. First, respondents were asked “Thinking about last week, were you or others in your household [un]able to eat healthy and nutritious food?” We coded the “Yes” or “No” responses in the affirmative if the respondents reported being unable to eat healthy or nutritious food. Respondents were then asked (Yes or No) “Still thinking about last week, was there a time when you or others in your household were hungry but did not eat?” These questions were first asked in April 2020 and then repeated in July 2020, allowing us to examine changing household food circumstances during the COVID-19 lockdown measures in the UK. It is important to note that instrument omitted a clause asking whether these experiences were due to resource constraints. For example, in the FIES, the corresponding questions are asked as follows: “Still thinking about the last 12 months, was there a time when you were unable to eat healthy and nutritious food because of a lack of money or other resources?”, and “Was there a time when you were hungry but did not eat because there was not enough money or other resources for food?” [9]. The Understanding Society COVID-19 web survey included a separate follow-up item referring probing reasons for respondents indicating that they were hungry but did not eat. Since this was not asked until the July wave, we were not able to include it in our analysis of changes in the prevalence of food-related hardships since the beginning of lockdown measures.

We analysed whether and how food-related hardships have changed during lockdown measures in the UK across key dimensions of social stratification. To capture pre-existing vulnerability to a macro-economic shock, we extracted household income from Wave 9 of the UKHLS, which was collected from 2017-2019, and linked this to respondents’ data from COVID-19 web survey. We also linked respondents’ highest qualification, because questions about educational attainment were not asked in the COVID-19 web survey. We used the following demographic characteristics from the April 2020 COVID-19 web survey: age, gender, race/ethnicity, cohabitation status, government office region, number of children aged 0-4 in the household, number of children aged 5-15 in the household, number of children aged 16-18 in the household. We also analysed changes in food intake perceptions according to employment status in both April and July using a derived variable constructed by the UKHLS team, which categorizes respondents into the “Employed,” “Self-employed,” “Both employed and self-employed,” or “Not employed.” We further separated the “Not employed” group into those below the age of 65, and those 65 and over. Table 1 provides descriptive statistics for all study variables.

**Table 1:** Descriptive Statistics (N = 11,104).

*Gender*	
Male	47.8%
Female	52.2%
*Race / Ethnicity*	
White	93.8%
Asian	3.6%
Black	1.5%
Mixed	1.1%
*Highest qualification*	
Degree	29.5%
Other higher degree	11.8%
A-level etc	21.5%
GCSE etc	21.3%
Other qualification	8.9%
No qualification	7.0%
*Age groups*	
16-24	6.2%
25-34	10.2%
35-44	14.3%
45-54	18.2%
55-64	22.0%
65-74	18.6%
75+	10.5%
*Living with a partner in April 2020*	
Yes	65.3%
No	34.7%
*Quintiles of household income (equivalized), 2017-19*	
Bottom (mean)	£ 911.94
2 (mean)	£ 1,452.77
3 (mean)	£ 1,867.40
4 (mean)	£ 2,384.65
Top (mean)	£ 4,085.14
*Number of children in the household - Aged 0-4in April 2020*	
0	92.0%
1	6.2%
2+	1.8%
*Number of children in the household - Aged 5-15in April 2020*	
0	79.5%
1	10.4%
2+	10.1%
*Number of children in the household - Aged 16-18in April 2020*	
0	91.4%
1	7.9%
2+	0.7%
*Government Office Region*	
North East	4.1%
North West	10.6%
Yorkshire and The Humber	8.8%
East Midlands	8.1%
West Midlands	9.2%
East of England	10.6%
London	10.4%
South East	14.7%
South West	9.4%
Wales	4.5%
Scotland	7.6%
Northern Ireland	2.0%
*Employment status (April 2020)*	
Employed	48.8%
Self-employed	6.6%
Both employed and self-employed	1.7%
Not employed, below 65	17.0%
Not employed, 65+	25.8%
*Employment status (July 2020)*	
Employed	51.0%
Self-employed	7.0%
Both employed and self-employed	2.0%
Not employed, below 65	17.5%
Not employed, 65+	22.3%

Note: Complex sampling design weights that adjust for representativeness and non-random attrition are taken into account.

For our second objective, we incorporate the corresponding derived employment status items from the May and July waves of the COVID -19 web survey study to identify a series of employment transitions among those who were employed at baseline, as assessed in a retrospective question relating to January / February 2020 (pre-lockdown). We first restricted our sample to non-retired respondents aged 20-64 who reported being employed, self-employed, or both employed and self-employed at baseline. We then constructed three groups. The first were in employment throughout the period January/February to July 2020 (n = 3,806). The second were either furloughed or eligible for the equivalent self-employment support scheme. This group comprises respondents who stopped working but remained in employment while being paid 80% of their previous income, up to a limit (up to a maximum salary of £2,500 per month for employees up to £7,500 for self-employed). These individuals reported employment in January/February, then reported being furloughed under the Coronavirus Job Retention Scheme or eligible for the self-employment support scheme in either April, May, June, OR July (N = 1,558). The third group transitioned from employed to out of employment without being furloughed or being eligible for the equivalent self-employment support scheme. They reported employment in January/February, then were no longer in employment in either April, May, June, OR July (N = 266). The third group transitioned from employed to out of employment without being furloughed or being eligible for the SEISS. They reported employment in January/February, then were no longer in employment in either April, May, June, OR July. Respondents in this last group did not indicate that they had been furloughed under the JRS or being eligible for the SEIS.

### 2.3 Statistical modelling

Our analyses unfold in two stages. First, to quantify changes in the prevalence of food-related hardships we calculate the mean and 95% confidence intervals for each outcome in April and July 2020 for our sample as a whole and stratified by the sociodemographic variables described above. Tables 2 and 3 report the prevalence of as well as the percentage point change from April to July.

**Table 2:** Prevalence of being unable to eat healthy and nutritious food across socioeconomic and demographic groups (N=11,104).

	Lack nutrition in April weighted %	95 % confidence intervals			Lack nutrition in July weighted %	95 % confidence intervals			Percentage point change
Total analytic sample	**3.2%**	2.2%	to	4.2%	**16.3%**	15.0%	to	17.7%	**13.1%**
*Gender*									
Male	**2.9%**	1.4%	to	4.3%	**15.9%**	13.9%	to	18.0%	**13.0%**
Female	**3.5%**	2.3%	to	4.8%	**16.7%**	15.0%	to	18.5%	**13.2%**
*Race / Ethnicity*									
White	**2.8%**	1.9%	to	3.7%	**15.6%**	14.2%	to	17.0%	**12.8%**
Asian	**5.2%**	-0.1%	to	10.4%	**28.5%**	20.4%	to	36.6%	**23.3%**
Black	**25.5%**	-4.3%	to	55.4%	**34.3%**	12.6%	to	56.1%	**8.8%**
Mixed	**2.5%**	-0.1%	to	5.1%	**11.9%**	4.8%	to	18.9%	**9.4%**
*2017-19 quintiles of household income (equivalized)*									
1	**6.5%**	3.0%	to	9.9%	**19.2%**	15.6%	to	22.7%	**12.7%**
2	**3.5%**	2.1%	to	5.0%	**16.8%**	14.3%	to	19.3%	**13.3%**
3	**2.7%**	1.4%	to	4.1%	**16.2%**	13.3%	to	19.2%	**13.5%**
4	**1.2%**	0.3%	to	2.2%	**15.5%**	12.6%	to	18.3%	**14.2%**
5	**0.8%**	0.2%	to	1.5%	**12.8%**	10.5%	to	15.1%	**12.0%**
*Highest qualification in 2017-19*									
Degree	**0.8%**	0.5%	to	1.2%	**15.7%**	13.5%	to	17.9%	**14.9%**
Other higher degree	**2.1%**	0.9%	to	3.2%	**14.9%**	12.2%	to	17.6%	**12.8%**
A-level etc	**4.2%**	1.5%	to	6.9%	**15.4%**	12.2%	to	18.6%	**11.2%**
GCSE etc	**4.7%**	1.9%	to	7.6%	**17.1%**	14.0%	to	20.3%	**12.4%**
Other qualification	**5.1%**	1.1%	to	9.0%	**19.7%**	15.9%	to	23.5%	**14.6%**
No qualification	**5.1%**	1.3%	to	8.9%	**18.4%**	11.3%	to	25.4%	**13.3%**
*Employment status*									
Employed	**2.3%**	1.5%	to	3.1%	**16.1%**	14.4%	to	17.8%	**13.8%**
Self-employed	**1.1%**	0.3%	to	1.9%	**16.8%**	11.6%	to	22.0%	**15.7%**
Both employed and self-employed	**3.7%**	-2.8%	to	10.3%	**17.8%**	10.3%	to	25.3%	**14.1%**
Not employed, below 65	**8.5%**	4.6%	to	12.3%	**19.9%**	15.3%	to	24.5%	**11.4%**
Not employed, 65+	**2.0%**	0.0%	to	4.0%	**13.7%**	11.7%	to	15.6%	**11.7%**
*Age groups*									**0.0%**
16-24	**4.7%**	1.5%	to	7.9%	**10.9%**	5.5%	to	16.3%	**6.2%**
25-34	**4.4%**	1.5%	to	7.3%	**16.4%**	12.4%	to	20.3%	**12.0%**
35-44	**3.0%**	1.6%	to	4.4%	**19.9%**	16.5%	to	23.3%	**16.9%**
45-54	**4.7%**	2.2%	to	7.1%	**18.6%**	15.7%	to	21.5%	**13.9%**
55-64	**2.9%**	0.6%	to	5.3%	**17.5%**	13.9%	to	21.1%	**14.5%**
65-74	**2.4%**	-0.3%	to	5.2%	**13.0%**	10.7%	to	15.2%	**10.5%**
75+	**0.9%**	-0.1%	to	1.9%	**14.4%**	11.3%	to	17.5%	**13.5%**
*Living with a partner*									
Yes	**1.4%**	0.8%	to	1.9%	**16.4%**	14.9%	to	17.8%	**15.0%**
No	**7.1%**	4.4%	to	9.8%	**16.3%**	13.5%	to	19.1%	**9.2%**
*Number of children in the household aged 0-4*									
0	**3.3%**	2.2%	to	4.3%	**16.2%**	14.8%	to	17.7%	**13.0%**
1	**1.1%**	0.1%	to	2.0%	**19.4%**	14.3%	to	24.5%	**18.3%**
2+	**8.7%**	-7.8%	to	25.3%	**11.9%**	5.2%	to	18.5%	**3.1%**
*Number of children in the household aged 5-15*									
0	**3.3%**	2.2%	to	4.4%	**15.8%**	14.3%	to	17.4%	**12.5%**
1	**2.4%**	0.4%	to	4.4%	**14.9%**	11.7%	to	18.1%	**12.5%**
2+	**3.4%**	-0.7%	to	7.4%	**21.7%**	17.3%	to	26.0%	**18.3%**
*Number of children in the household aged 16-18*									
0	**3.2%**	2.1%	to	4.2%	**16.5%**	15.0%	to	17.9%	**13.3%**
1	**2.2%**	-0.1%	to	4.5%	**15.4%**	11.0%	to	19.8%	**13.2%**
2+	**21.0%**	-13.9%	to	55.8%	**14.7%**	-1.0%	to	30.4%	**-6.2%**
*Government Office Region*									
North East	**5.6%**	-1.4%	to	12.6%	**10.9%**	6.6%	to	15.2%	**5.4%**
North West	**1.8%**	0.5%	to	3.1%	**16.5%**	12.6%	to	20.4%	**14.7%**
Yorkshire and The Humber	**4.4%**	0.2%	to	8.7%	**14.2%**	10.6%	to	17.8%	**9.8%**
East Midlands	**1.8%**	0.4%	to	3.2%	**17.4%**	13.6%	to	21.2%	**15.6%**
West Midlands	**3.5%**	2.1%	to	4.8%	**16.9%**	10.9%	to	22.9%	**13.4%**
East of England	**0.3%**	0.0%	to	0.5%	**13.8%**	9.8%	to	17.9%	**13.6%**
London	**6.6%**	1.9%	to	11.3%	**22.2%**	17.3%	to	27.2%	**15.7%**
South East	**2.8%**	0.5%	to	5.0%	**15.5%**	12.0%	to	19.0%	**12.7%**
South West	**4.4%**	1.2%	to	7.7%	**12.5%**	9.8%	to	15.2%	**8.1%**
Wales	**1.6%**	0.1%	to	3.0%	**15.2%**	8.9%	to	21.6%	**13.7%**
Scotland	**3.7%**	-2.6%	to	10.1%	**23.2%**	16.8%	to	29.5%	**19.4%**
Northern Ireland	**3.2%**	0.1%	to	6.3%	**12.7%**	7.1%	to	18.3%	**9.5%**

Note: Complex sampling design weights that adjust for representativeness and non-random attrition are taken into account.

**Table 3:** Prevalence of being hungry but not eating across socioeconomic and demographic groups (N=11,104).

	Hunger in April weighted %	95 % confidence intervals			Hunger in July weighted %	95 % confidence intervals			Percentage point change
Total analytic sample	**3.3%**	2.5%	to	4.2%	**5.1%**	4.2%	to	6.0%	**1.8%**
*Gender*									
Male	**2.9%**	2.1%	to	3.7%	**4.9%**	3.4%	to	6.4%	**2.0%**
Female	**3.7%**	2.3%	to	5.1%	**5.3%**	4.2%	to	6.4%	**1.5%**
*Race / Ethnicity*									**0.0%**
White	**3.2%**	2.3%	to	4.1%	**4.9%**	4.0%	to	5.9%	**1.7%**
Asian	**4.9%**	1.3%	to	8.6%	**7.4%**	0.3%	to	14.5%	**2.5%**
Black	**3.1%**	0.8%	to	5.3%	**5.5%**	0.3%	to	10.6%	**2.4%**
Mixed	**9.5%**	3.2%	to	15.8%	**10.5%**	2.3%	to	18.6%	**1.0%**
*2017-19 quintiles of household income (equivalized)*									
1	**6.6%**	3.6%	to	9.6%	**8.4%**	5.5%	to	11.2%	**1.8%**
2	**4.1%**	2.6%	to	5.6%	**5.9%**	3.9%	to	7.8%	**1.8%**
3	**2.3%**	1.2%	to	3.3%	**4.7%**	3.0%	to	6.4%	**2.4%**
4	**1.3%**	0.6%	to	2.0%	**2.1%**	1.2%	to	3.0%	**0.9%**
5	**1.2%**	0.4%	to	2.0%	**3.2%**	1.7%	to	4.7%	**2.0%**
*Highest qualification in 2017-19*									
Degree	**1.6%**	1.0%	to	2.3%	**3.7%**	1.9%	to	5.5%	**2.0%**
Other higher degree	**3.0%**	1.3%	to	4.7%	**4.3%**	2.6%	to	5.9%	**1.3%**
A-level etc	**3.7%**	2.4%	to	5.0%	**6.1%**	3.8%	to	8.4%	**2.4%**
GCSE etc	**3.6%**	2.5%	to	4.7%	**5.5%**	4.1%	to	7.0%	**1.9%**
Other qualification	**4.0%**	2.3%	to	5.8%	**4.2%**	2.2%	to	6.3%	**0.2%**
No qualification	**8.2%**	-0.9%	to	17.3%	**9.1%**	4.2%	to	14.1%	**0.9%**
*Employment status*									
Employed	**2.5%**	1.8%	to	3.3%	**4.5%**	3.5%	to	5.5%	**2.0%**
Self-employed	**2.9%**	1.1%	to	4.7%	**5.3%**	2.4%	to	8.2%	**2.4%**
Both employed and self-employed	**3.4%**	-2.9%	to	9.7%	**3.1%**	0.0%	to	6.2%	**-0.3%**
Not employed, below 65	**9.7%**	5.7%	to	13.8%	**10.9%**	7.6%	to	14.2%	**1.2%**
Not employed, 65+	**0.8%**	0.4%	to	1.1%	**2.0%**	0.1%	to	3.9%	**1.2%**
*Age groups*									
16-24	**8.5%**	4.9%	to	12.1%	**9.4%**	5.6%	to	13.3%	**0.9%**
25-34	**9.4%**	5.0%	to	13.8%	**7.0%**	5.0%	to	9.1%	**-2.4%**
35-44	**3.0%**	1.6%	to	4.5%	**4.9%**	2.9%	to	7.0%	**1.9%**
45-54	**4.0%**	2.2%	to	5.9%	**6.2%**	4.1%	to	8.3%	**2.2%**
55-64	**2.1%**	1.3%	to	2.9%	**5.1%**	2.7%	to	7.5%	**3.0%**
65-74	**0.9%**	0.4%	to	1.4%	**1.3%**	0.8%	to	1.9%	**0.4%**
75+	**0.5%**	0.2%	to	0.8%	**3.1%**	-1.5%	to	7.6%	**2.6%**
*Living with a partner*									
Yes	**2.4%**	1.8%	to	2.9%	**3.2%**	2.6%	to	3.8%	**0.8%**
No	**5.4%**	3.1%	to	7.7%	**8.8%**	6.4%	to	11.2%	**3.4%**
*Number of children in the household aged 0-4*									
0	**3.3%**	2.4%	to	4.2%	**5.3%**	4.3%	to	6.3%	**1.9%**
1	**2.9%**	0.1%	to	5.7%	**3.3%**	1.6%	to	5.0%	**0.4%**
2+	**5.0%**	1.2%	to	8.7%	**3.8%**	0.7%	to	6.9%	**-1.2%**
*Number of children in the household aged 5-15*									
0	**2.6%**	2.1%	to	3.2%	**5.1%**	4.0%	to	6.2%	**2.5%**
1	**3.9%**	1.8%	to	6.0%	**4.6%**	2.7%	to	6.4%	**0.7%**
2+	**8.3%**	2.0%	to	14.6%	**5.5%**	3.3%	to	7.8%	**-2.7%**
*Number of children in the household aged 16-18*									
0	**3.4%**	2.5%	to	4.3%	**5.2%**	4.2%	to	6.3%	**1.9%**
1	**2.7%**	0.5%	to	5.0%	**4.0%**	1.9%	to	6.2%	**1.3%**
2+	**3.6%**	-4.2%	to	11.4%	**0.6%**	-0.2%	to	1.3%	**-3.1%**
*Government Office Region*									
North East	**2.6%**	-0.5%	to	5.6%	**2.7%**	-0.5%	to	5.8%	**0.1%**
North West	**2.9%**	1.4%	to	4.3%	**7.3%**	2.4%	to	12.1%	**4.4%**
Yorkshire and The Humber	**2.7%**	0.4%	to	5.0%	**4.5%**	2.3%	to	6.7%	**1.8%**
East Midlands	**5.2%**	3.4%	to	7.0%	**4.7%**	2.9%	to	6.6%	**-0.5%**
West Midlands	**4.5%**	2.5%	to	6.4%	**5.2%**	3.6%	to	6.9%	**0.7%**
East of England	**2.7%**	0.8%	to	4.5%	**4.9%**	2.3%	to	7.4%	**2.2%**
London	**3.0%**	2.1%	to	3.9%	**6.1%**	3.6%	to	8.6%	**3.1%**
South East	**4.3%**	-0.3%	to	9.0%	**4.8%**	2.6%	to	7.0%	**0.5%**
South West	**2.9%**	0.9%	to	4.9%	**3.6%**	2.0%	to	5.2%	**0.7%**
Wales	**1.0%**	0.1%	to	2.0%	**5.0%**	-0.4%	to	10.3%	**3.9%**
Scotland	**2.9%**	1.6%	to	4.2%	**6.1%**	0.7%	to	11.4%	**3.2%**
Northern Ireland	**4.6%**	1.4%	to	7.7%	**4.0%**	0.7%	to	7.3%	**-0.6%**

Note: Complex sampling design weights that adjust for representativeness and non-random attrition are taken into account.

**Fig. 2:**
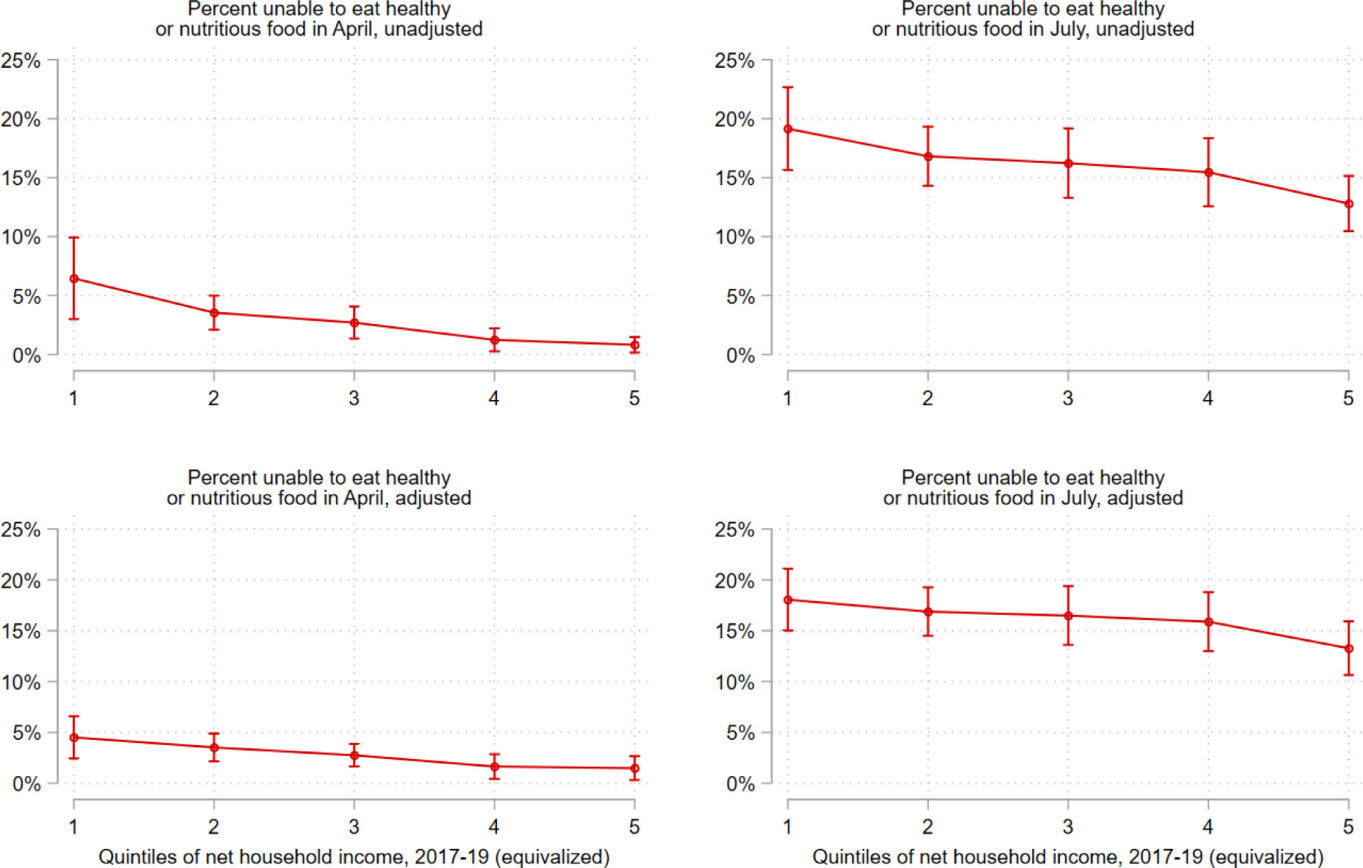
**Shift in the probability of being unable to eat healthy and nutritious food across quintiles of household incomes in 2017-19 (equivalized), April-July 2020** Note: Estimates and 95% confidence intervals are marginal effects derived from logistic regression models. Adjusted models (bottom panel) control for age, gender, race/ethnicity, number of children at home, highest qualification in 2017-19, employed status, and geographic region. Complex sampling design weights that adjust for representativeness and non-random attrition are taken into account.

**Fig. 3:**
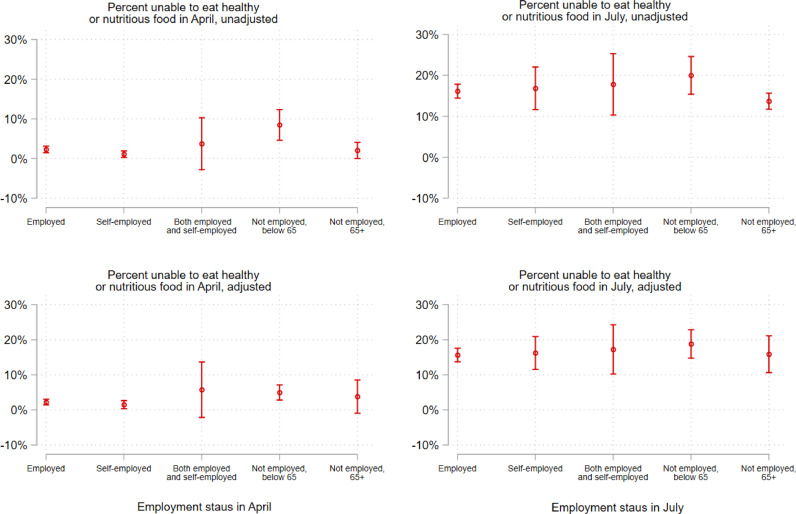
**Shift in the probability of being unable to eat healthy and nutritious food by employment status, April-July 2020** Note: Estimates and 95% confidence intervals are marginal effects derived from logistic regression models. Adjusted models (bottom panel) control for age, gender, race/ethnicity, number of children at home, highest qualification in 2017-19, equivalized household income in 2015-17, and geographic region. Complex sampling design weights that adjust for representativeness and non-random attrition are taken into account.

Fig. 2, Fig. 3 illustrate the association between each food-related hardship item and 1) household income, and 2) employment status in April and July. Here, we present the unadjusted (upper panel) and adjusted (lower panel) association between both outcome variables and household income (Fig. 2) and employment status (Fig. 3). Estimates and 95% confidence intervals are marginal effects derived from logistic regression models (Stata's *logit* command) to. Adjusted models (bottom panel) in Fig. 2 control for age, gender, race/ethnicity, number of children at home, highest qualification in 2017-19, employed status, and geographic region. Adjusted models (bottom panel) in Fig. 3 control for age, gender, race/ethnicity, number of children at home, highest qualification in 2017-19, equivalized household income in 2015-17, and geographic region.

**Fig. 4:**
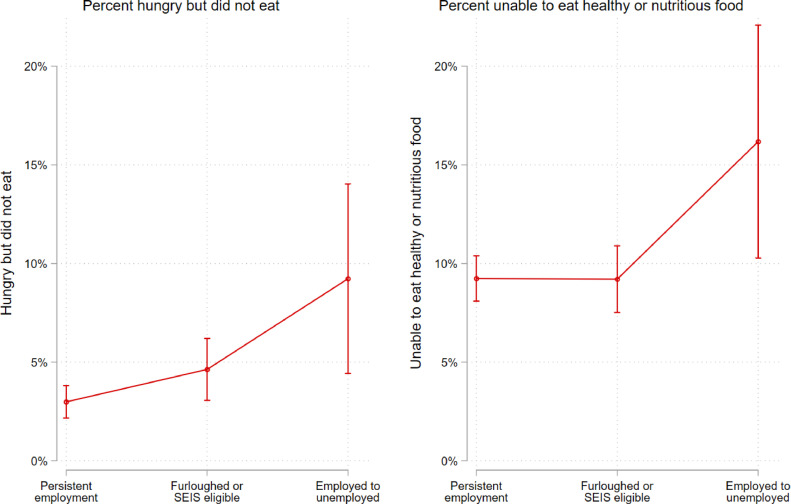
**Association between labour force transitions and food-related hardships in April and July (N = 5,630), fully adjusted, all respondents employed at baseline (January/February).** Note: Estimates and 95% confidence intervals are marginal effects derived from pooled logistic regression models that control for age, gender, race/ethnicity, number of children at home, highest qualification in 2017-19, equivalized household income in 2015-17, and geographic region. Complex sampling design weights that adjust for representativeness and non-random attrition are taken into account. All individuals in this sample were employed at baseline (January or February 2020) and then were either still employed, furloughed or eligible for the SEISS, or unemployed in any month from April through July 2020. The y-axis is the probability of being hungry but not eating in the past week (left) or being unable to eat healthy and nutritious food in the past week (right) in either April or July.

Fig. 4 illustrates the relationship between labour force transitions and food-related hardships in either April or July. Estimates and 95% confidence intervals are marginal effects derived from pooled logistic regression models that control for age, gender, race/ethnicity, number of children at home, highest qualification in 2017-19, equivalized household income in 2015-17, and geographic region. All individuals in this sample were employed at baseline (January or February 2020) and then were either still employed, furloughed or eligible for the SEISS, or unemployed in any month from April through July 2020. The y-axis is the probability of being hungry but not eating in the past week (left) or being unable to eat healthy and nutritious food in the past week (right) in either April or July. All figures use Mize's graphics scheme *cleanplots* in Stata [23]*.*

### 2.4 Role of the funding source

The funders had no role in the study design, data collection, data analysis, interpretation, the writing of the report or decisions on where to publish.

## 3 Results

Unadjusted prevalence estimates for changes in reports of being unable to eat health and nutritious food between April and July are described in Table 2. There has been a marked rise in these reports during the pandemic. In April 2020, 3•2% of respondents reported being unable to eat healthy and nutritious food in the last week. By July, this had increased to 16•3%, a 13•1-percentage point increase, but varied substantially within the population. Some of the largest increases were seen among Asian respondents (23•3 percentage points), the self-employed (15•7 percentage points) respondents aged 35-44 (16•9 percentage points), those with two or more children aged 5-15 in the household or with one child aged below four (18•3 percentage points) and those living in Scotland, London, and the North West of England and Midlands (19•4 percentage points, 15•7 percentage points, and 14•7 percentage points, 15•6 percentage points for East Midlands and 13•4 percentage points for West Midlands respectively). While changes in reported inability to eat healthy and nutritious food were similar across income groups, Table 2 shows a clear gradient in prevalence in both April and July. The highest prevalence is observed among respondents in the bottom quintile of equivalized household income, followed by the second quintile, and so on.

Fig. 2, Fig. 3 illustrate inequalities and shifts in the prevalence of reported inability to eat healthy and nutritious food across key socio-demographic groups, presenting both unadjusted and adjusted estimates. In April, these reports were, as expected, higher among those in the lowest quintile of household income (Fig. 2), although the gradient diminished in the fully adjusted model and no statistically significant differences can be found among the highest four quintiles. By July, the probability of reporting being unable to eat healthy and nutritious food had increased by more than twofold for all quintiles, with gradients in the adjusted and unadjusted model similar to those in April. Looked at by employment status, again, as expected, reported inability to eat healthy and nutritious food was greatest among those who were below age 65 and not employed, and very low for those in any form of employment (Fig. 2). Again, by July, this had increased markedly for all groups.

Unadjusted prevalence estimates for reports of being hungry but not eating in the last week between April and July are described in Table 3. These reports increased 1•8-percentage points from April 2020 (3•3%) to July (5•1%). While the changes shown in Table 3 were smaller than those observed for reported inability to eat healthy and nutritious food, the prevalence of being hungry but not eating was clearly concentrated in more disadvantaged groups. The highest prevalence of these reports is observed among respondents in the bottom quintile of equivalized household income (6•6% and 8•4% in April and July respectively), while the lowest prevalence is observed among those in the highest quintile (1•2% and 3•2% in April and July respectively). Similarly, the prevalence of being hungry but not eating is highest among those with no qualifications (8•2% and 9•1% in April and July respectively) and lowest among those with a degree (1•6% and 3•7% in April and July respectively). Finally, across all sociodemographic characterises included in this study in July, the highest overall prevalence of being hungry but not eating was observed among those not employed below age 65 (10•9%).

Turning to those experiencing employment transitions (Fig. 4), those moving from employment to unemployment had higher odds of being hungry but not eating in the last week relative to furloughed individuals (OR = 2•2; p < 0•05; 95% CI: 1•1 to 4•2) and to the persistently employed (OR = 3•5; p < 0•001; 95% CI: 1•8 to 6•9), adjusting for age, highest qualification in 2017-19, net household income in 2017-19 (equivalized), gender, race/ethnicity, number children at home (aged 0-4, 5-15, and 16-18), cohabitation status, and government office region. Respondents moving from employment to unemployment also had higher odds of reporting an inability to eat healthy and nutritious food relative to furloughed individuals (OR = 1•9; p < 0•05; 95% CI: 1•4 to 3•2) and to the persistently employed (OR = 2•0; p < 0•01; 95% CI: 1•2 to 3•4).

Importantly, furloughed individuals and those eligible for the equivalent self-employment scheme did not differ according to statistically significant thresholds in their probability of being hungry but not eating compared to the persistently employed (OR = 1•6; p = 0•06; 95% CI: 0•98 to 2•6). Similarly, we did not observe statistically significant differences between furloughed individuals and the persistently employed in their probability of reporting an inability to eat healthy and nutritious food (OR = 1•0; p = 0•7; 95% CI: 0•8 to 1•3). Fig. 4 depicts these associations visually, and Table A3 in the appendix presents these point estimates and confidence intervals.

### 3.1 Sensitivity analyses

**Fig. A1:**
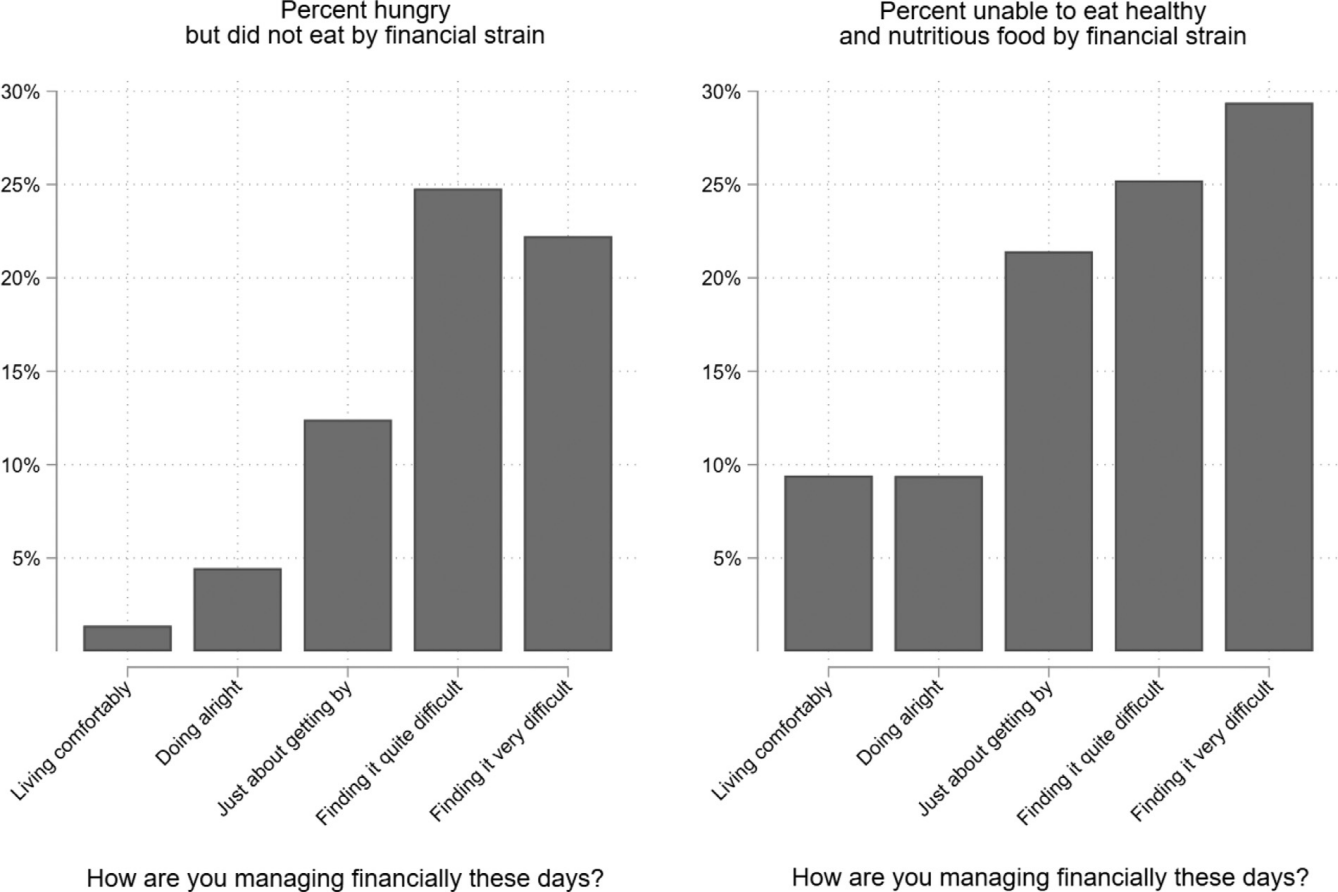
**Prevalence of food-related hardships across levels of financial strain, pooled logistic regression models from the April and July waves (pooled) of Understand Society COVID-19 web survey** Note: Estimates incorporate weights used for complex survey design, representativeness, and attrition.

Our analyses of changes in the prevalence of self-reported lack of nutrition and hunger revealed similar increases in these perceptions across socio-demographic such as income and education, which was unexpected. We therefore conducted supplementary analyses of the bivariate association between financial strain and both outcomes in order to confirm prior research that finds deteriorating financial circumstances to be a strong predictor of food insecurity, and to provide evidence that our measures are serving as an adequate proxy for food insecurity. Fig. A1 in the appendix illustrates these relationships, showing subjective financial strain to be a strong determinant of self-reported lack of nutrition and hunger.

**Fig. A2:**
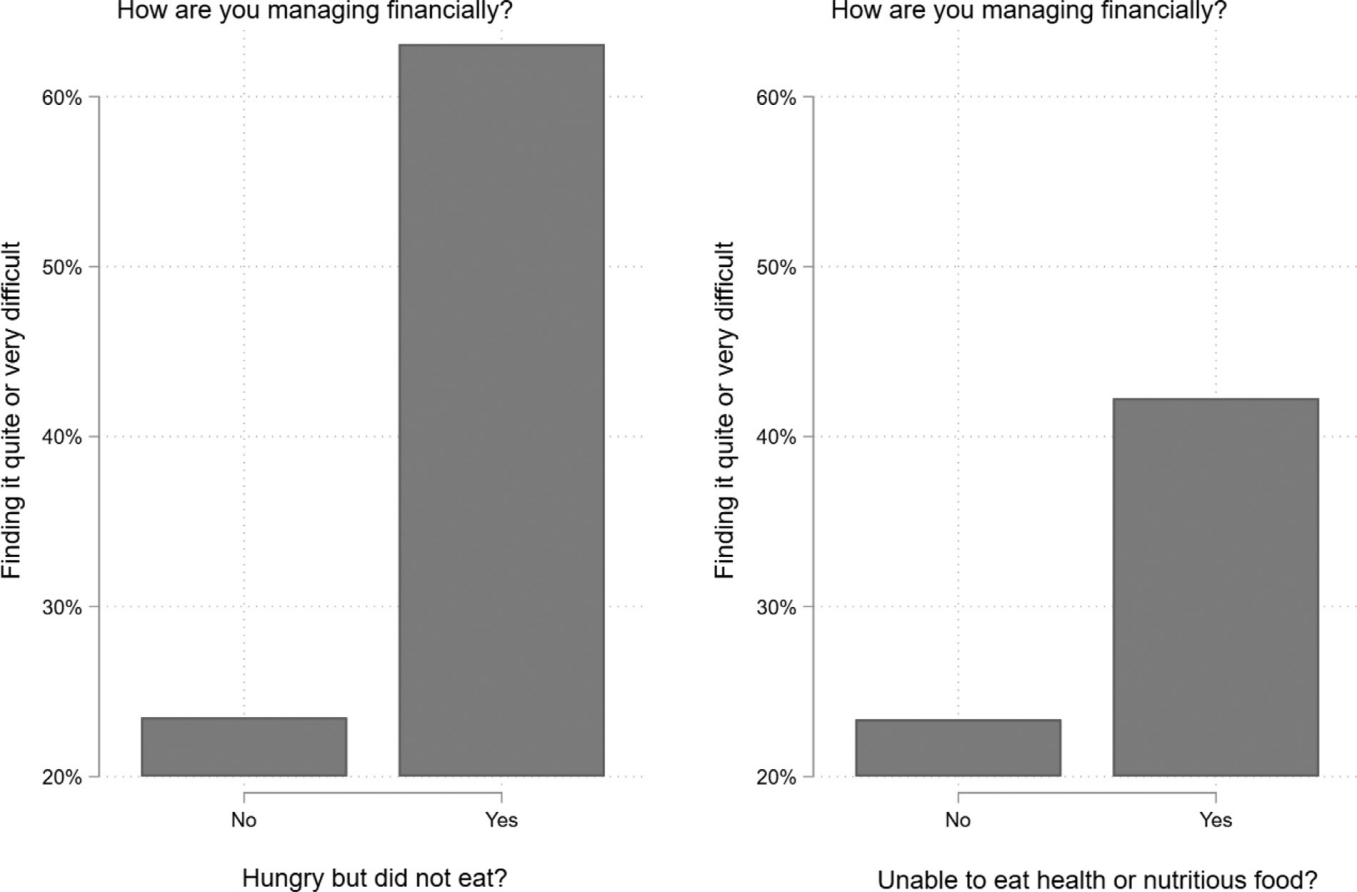
**Food-related hardships predicting finding it quite or very difficult to manage financially these days, logistic regression models from the April and July waves (pooled) of Understanding Society COVID-19 web survey** Note: Estimates incorporate weights used for complex survey design, representativeness, and attrition. Respondents were asked, “How well would you say you yourself are managing financially these days?” With responses coded “Living comfortably [1]”, “Doing alright [2]”, Just about getting by [3]”, “Finding it quite difficult [4]”, and “Finding it very difficult [5]”. We created a dichotomous variable, with [1] representing all respondents indicating that they are “Finding it quite difficult”, OR “Finding it very difficult”. All other response categories were coded (0).

Conversely, Fig. A2 shows the proportion of respondents who answered in the affirmative to being hungry but not eating and being unable to eat health and nutritious food who also reported being in stained financial circumstances. Here we dichotomize subjective financial strain. In the original question, respondents were asked, “How well would you say you yourself are managing financially these days?” With responses coded “Living comfortably [1]”, “Doing alright [2]”, Just about getting by [3]”, “Finding it quite difficult [4]”, and “Finding it very difficult [5]”. We created a dichotomous variable, with [1] representing all respondents indicating that they are “Finding it quite difficult”, OR “Finding it very difficult”. All other response categories were coded (0). Fig. A2 shows that approximately 63% of respondents who reported being hungry but not eating are also finding it quite or very difficult to manage financially, while 42% of respondents who reported being unable to eat healthy or nutritious food are also finding it quite or very difficult to manage financially.

We also conducted additional analyses to determine which subgroups were most likely to have excluded from our sample because they did not respond to both food-related hardships items in both April and July—our first sample restriction criteria. It may be the case, for example, that more vulnerable groups dropped out of our sample, which would likely bias our estimates for those groups in the conservative direction. As noted in the description of our data, we restricted our sample to those with non-missing responses on both outcomes. Of those included in the first wave of the Understanding Society COVID-19 web survey (N = 17,452), we created a dichotomous variable for missingness, coded “1” if participants did not respond to both dependent variables in both April and July (N = 5,295), and “0” if respondents did respond to both items in both waves (N = 12,157). We tested whether those respondents with missing values for our outcome measures in both April and July differed from those who did not, by all study variables. Table A1 in the appendix presents a logistic regression model predicting missingness on food-related hardships in both April and July (N = 11,234 after removing missing values on all predictors). Here, we find that the self-employed in April are less likely to be missing relative to the employed, while those with 2 or more children aged 5 to 15 are more likely to be missing compared to those with no children in that age group. Asian respondents were 2•9 times more likely than white respondents to be missing, and Black and mixed ethnicity respondents were 6•3 and 5•3 times more likely than whites to be missing (although coefficients for these latter two groups did not reach statistical significance, likely because of small cell sizes). We also detect some regional variations, with those in Scotland, Wales, and the North East of England more likely to have missing responses compared to those residing in London.

**Table A1:** Logistic regression model predicting missingness on both food-related hardship items in both April and July.

	Odds Ratio	95% CI		
Female (ref: male)	1.10	0.58	to	2.09
*Race (ref: white)*				
Asian	2.88	1.14	to	7.23
Black	6.32	0.68	to	58.7
Mixed	5.32	0.95	to	29.8
*Equivalized household income quintiles in 2017-19*				
*(ref: bottom quintile)*				
2nd	0.57	0.21	to	1.58
3rd	0.59	0.23	to	1.51
4th	0.42	0.16	to	1.06
5th	0.44	0.16	to	1.21
*Highest qualification in 2017-19 (ref: degree)*				
Other higher degree	2.08	0.92	to	4.70
A-level etc	0.47	0.21	to	1.07
GCSE etc	1.08	0.44	to	2.64
Other qualification	0.81	0.23	to	2.80
No qualification	0.71	0.16	to	3.06
*Age groups (ref: 16-24)*				
25-34	1.20	0.31	to	4.61
35-44	0.79	0.23	to	2.73
45-54	0.60	0.19	to	1.84
55-64	0.32	0.08	to	1.28
65-74	1.49	0.21	to	10.5
75+	0.69	0.12	to	4.02
Living alone (ref: living with a partner)	1.34	0.75	to	2.4
*Number of school aged children in the household - Aged 0-4 (ref: none)*				
1	1.17	0.38	to	3.56
2+	0.06	0.00	to	2.84
*Number of school aged children in the household - Aged 5-15 (ref: none)*				
1	1.48	0.7	to	3.16
2+	4.47	1.92	to	10.4
*Number of school aged children in the household - Aged 16-18 (ref: none)*				
1	0.95	0.33	to	2.76
2+	2.60	0.44	to	15.4
*Government Office Region (ref: London)*				
North East	6.79	1.57	to	29.3
North West	1.73	0.5	to	6.01
Yorkshire and The Humber	1.62	0.36	to	7.25
East Midlands	2.00	0.43	to	9.37
West Midlands	0.74	0.15	to	3.69
East of England	1.64	0.31	to	8.56
South East	1.74	0.44	to	6.85
South West	2.61	0.56	to	12.1
Wales	5.12	1.33	to	19.8
Scotland	5.11	1.21	to	21.6
Northern Ireland	3.49	0.74	to	16.5
*Employment status in April (ref: Employed)*				
Self-employed	0.35	0.13	to	0.95
Both employed and self-employed	0.14	0.011	to	1.75
Not employed	0.47	0.13	to	1.73
*Employment status in July (ref: Employed)*				
Self-employed	1.54	0.56	to	4.29
Both employed and self-employed	4.30	0.72	to	25.6
Not employed	0.97	0.33	to	2.83

Note: Estimates incorporate weights used for complex survey design, representativeness, and attrition. The dependent variable represents those missing on both food-related hardships in either April or July. N = 11,234

**Table A2:** Links to study materials.

Understanding Society main survey	https://www.understandingsociety.ac.uk/documentation/mainstage
Understanding Society COVID-19 survey	https://www.understandingsociety.ac.uk/documentation/covid-19
User guide	https://www.understandingsociety.ac.uk/documentation/covid-19/user-guide
Participant communication materials	
for the web surveys	https://www.understandingsociety.ac.uk/sites/default/files/downloads/documentation/covid-19/fieldwork-documents/covid-19-communication-materials.pdf

**Table A3:** Logistic regression models predicting food-related hardships in April or July, N = 5,630.

	Hungry but did not eat		Unable to eat healthy and nutritious food	
	Odds Ratio	95 % CI	Odds Ratio	95 % CI
*Employment transitions (ref: persistently employed)*				
Employed to Furloughed or Self-Employment Income Support Scheme eligible	1.60	0 .98 to 2.63	1.00	0.77 to 1.29
Employed to Unemployed	3.49 ***	1.77 to 6.91	2.02 **	1.21 to 3.36

Note: Odds ratios and 95% confidence intervals are derived from pooled logistic regression models that control for age, gender, race/ethnicity, number of children at home, highest qualification in 2017-19, equivalized household income in 2015-17, survey month, and geographic region. Complex sampling design weights that adjust for representativeness and non-random attrition are taken into account. All individuals in this sample were employed at baseline (January or February 2020) and then were either still employed, furloughed or eligible for the SEISS, or unemployed in any month from April through July 2020. p<0.05; ** p<0.01; *** p<0.001.

## 4 Discussion

These findings document an alarming increase in food-related hardships, largely driven by increasing reports of being unable to eat healthy and nutritious food in the UK during the pandemic. Problems were anticipated [16] and measures were taken to mitigate them. However, it is clear that, between April and July 2020, they have had limited success.

The number of people reporting lacking health and nutritious food has increased five-fold. In July, roughly 11% of not employed individuals under the age of 65 reporting being hungry but not eating in the past week. All of the groups examined in this analysis have been affected, but some more than others.

Our findings are consistent with a recent report, using different data and food insecurity measures, which found that adults who were working in February 2020 but who reported transitioning to unemployment in May or July were about 2•5 times more likely to experience food insecurity compared to those who remained employed (18•5% vs. 7•4%, respectively) [17]. The same study did not find a similar increase for those who had been working in February but who were furloughed in May or June, also suggesting this scheme has mitigated what would have otherwise been a more substantial rise in food insecurity among this group. However, unlike in our study, furloughed respondents reported significantly higher rates of food insecurity relative to those who remained employed. The present study also corroborates findings from a cross-sectional study conducted in early April, again using different data and food insecurity measures, that found higher rates of food insecurity in the UK relative to 2018 [18]. Importantly this latter study relied on items used to measure food insecurity that referred to a 12-month time span in 2018 and then span a 30-day time span in April 2020, a potential source of bias for examining changes in population prevalence over time. Finally, our study is consistent with recent findings from the USA, finding that food insufficiency among all adults increased five-fold during the COVID pandemic compared to 2019, with African-Americans hardest hit [19].

As with all analyses of survey data, our study has several limitations. First, our analysis does not provide a causal explanation of the impact of COVID on food-related hardships, just an association. Second, the COVID web survey includes the clause to determine that the compromises in intakes were “because of a lack of money or other resources” only starting from July, therefore we cannot rely on a standardized, validated measure of food insecurity. Although we believe that the highlighted rise in food-related hardships can provide a useful discussion on the topic, the omission of these standardized measures is a serious limitation that complicates the interpretability of our findings. We recommend the inclusion of the standardized measures of food insecurity in the survey moving forward. Third, the COVID web wave was only able to link data to 40% of respondents from wave 8 or 9 of the UKHLS. This does not affect internal validity, but complicates assessing representativeness of the UK population. To address this, sampling weights were employed. Further we tested whether those respondents with missing values for food-related hardships in both April and July differed from those who did not by all study variables. Table A1 in the appendix presents a logistic regression model predicting missingness on food hardships in both April and July. Here, we find that the self-employed in April are less likely to be missing relative to the employed, while those with 2 or more children aged 5 to 15 are more likely to be missing compared to those with no children in that age group. Asian respondents were 2•9 times more likely than white respondents to be missing, and Black and mixed ethnicity respondents were 6•3 and 5•3 times more likely than whites to be missing (although coefficients for these latter two groups did not reach statistical significance, likely because of small cell sizes). We also detect some regional variations, with those in Scotland, Wales, and the North East of England more likely to be missing compared to those residing in London. Fourth, our results do not explicitly identify the disparate factors that might play a role in the associations reported here, such as lockdowns measures intended to mitigate the spread of the virus, or variations in the supply of food, which have been associated with increased food insecurity [18, 20]. Fifth, we only examine the impact of moving from employment to out of employment (furloughed or not), but we do not examine the impact of reducing the number of working hours or reasons for doing so, either voluntarily or not, which might lead to a reduced income and hence to an increased risk of becoming food insecure. Sixth, our analysis is based on food-related hardships among individuals included in the survey. This does not capture the experience of vulnerable groups, such as children and homeless people, for which future research is urgently needed. Finally, our outcome measures do not provide information about the nature and duration of dietary compromise indicated and therefore we cannot judge the seriousness of the comprised nutritional intakes and health implications derived from them.

We observed similar absolute increases in reports of being unable to eat healthy and nutritious food across income groups. We were unable to differentiate the reasons for this. However, one plausible explanation among high income groups is that these rises were observed not due to financial resource constraints but to available household food supplies and constrained shopping behaviours. It is also important to highlight the overall levels of food-related hardships in both April and July, which suggest a clear gradient, with lower income individuals still reporting these perceptions to the highest degree in both waves. In order to confirm prior research that finds deteriorating financial circumstances to be a strong predictor of food insecurity, and to provide evidence that our measures are serving as a reasonable proxy for food insecurity, we also performed a sensitivity check by assessing the association between financial strain and both outcomes in this study. Here, consistent with the hypothesis that these experiences correlate with economic hardship, we show subjective financial strain to be a strong determinant of food-related hardships. We also wish to point out that our results for changes in food-related hardships among low-income groups are likely conservative. Vulnerable individuals who had the highest propensity to experience increasing food-related hardships from April to July are likely the same individuals who dropped out of the survey between waves, which would downwardly bias our estimates.

Reports of being hungry but not eating and an inability to eat health and nutritious food may represent an entry point to investigate food insecurity. They may reflect deteriorating household economic circumstances or, in the unique case of the coronavirus pandemic, a reduced food supply. As noted in the introduction, there were shortages of food in shops immediately after the lockdown in March 2020, largely due to panic buying. Loopstra and colleagues found that a lack of food in shops alone explained about 40% of food insecurity experiences since the COVID-19 lockdown, and that 21•6% of adults reported feeling very worried or fairly worried about getting the food they need during the COVID-19 outbreak [18]. While food supply issues were rapidly resolved, the macroeconomic shock persisted. The number of people claiming unemployment related benefits increased three-fold during our study period, from 1•2 million in March to 2•7 million in July [21]. Employment protection schemes protected many households, but an estimated 2.9 million individuals slipped through the cracks, ineligible for the Job Retention Scheme or the Self-Employment Income Support Scheme.

A report for the UK Food Standards Agency offers some qualitative insights into the lived experiences of those affected [22]. The authors interviewed 20 UK citizens in June 2020, half of whom had been food insecure before the lockdown while the remainder became so after it was implemented. They describe how food insecurity and the need to respond to COVID-19, were superimposed upon many other challenges, including job insecurity, health issues, domestic violence, and debt. Factors contributing to increased risk and vulnerability included an inability to build or draw on financial safety nets, the lack of reliable full-time salaries, working in sectors that did not permit remote working, caring responsibilities that limited alternative sources of income, health, and particularly mental health challenges, and domestic abuse. Restrictions associated with the pandemic contributed to food insecurity in several ways. These included the loss of the inability to join family members for particular meals, such as Sunday lunches, that had previously helped them to stretch their budgets, an inability to afford supermarket delivery fees, reduced access to low-cost shops, competition for low-cost “value” brands that were especially likely to be stockpiled, price increases in shops serving deprived areas, and relying on others to help with their shopping, where a feeling of shame prevented them from asking for the cheapest brands to be purchased. The result was that those interviewed reported relying on food of poor nutritional quality, especially from tins or simple carbohydrates, skipping meals, and compromising on food safety by using out of date products. They also reported emotional problems, linked to the loss of family mealtimes. There was considerable awareness of food banks and food box schemes, but low uptake due to the associated stigma. Statutory welfare provisions, including both pre-existing ones, such as Universal Credit, and those introduced as part of the COVID response were reported as often being difficult to access.

Our results have important implications for policy. First, they demonstrate that the Coronavirus Job Retention scheme and the equivalent self-employment scheme appeared to have conferred protection against exposure to food-related hardships. However, many employees have been unable to benefit from the schemes, and it is unclear for how long it will be extended. Second, we show that all of the groups we examined have been affected. This is not a problem limited to those on the margins of society, although those at the margins suffer to a higher degree, however there is a clear gradient with lower income individuals being the hardest hit. We found evidence of the association between financial strain and both outcome variables in this study (Fig. A1). Third, our results are consistent with the hypothesis that, in practical terms, access to affordable food has declined. All age groups, including those of pensionable age whose incomes have largely been unaffected, have reported increases in their inability to eat healthy and nutritious food. Pandemic planning must include a component that addresses potential difficulties in food affordability and supply, with the precise responses tailored to the problems identified. These may range for general measures to replace lost income to targeted ones that address the needs of those who struggle to obtain access to food outlets, either physically or digitally.

Patient consent for publication: Not applicable

## Author Contributions

JK created the study, conducted the analysis, and wrote the first draft of the article. VT helped analyze the data and write the article. DS and MM oversaw the design of the study, facilitated interpretation of the findings, and helped write the article. All authors edited the final draft of the article. The corresponding author attests that all listed authors meet authorship criteria and that no others meeting the criteria have been omitted.

## Data Sharing Statement

The data used are publicly available via UK Data Service repository (study numbers 6614 and 8644) and do not require ethical assessment for academic research purposes.

Funding

DS is funded by the Wellcome Trust investigator award. JK and DS are funded by the European Research Council n. 313590 – HRES. VT is funded by the European Research Council n. 694145- IFAMID.

## Declaration of Competing Interest

None to declare. All authors have completed the ICMJE uniform disclosure form at www.icmje.org/coi_disclosure.pdf. DS and JK are supported by the the European Research Council 313590 - HRES. DS is also supported by the Wellcome Trust investigator award. VT is funded by the European Research Council, 694145- IFAMID.

## Appendix

Table A1

## Appendix B Supplementary materials

Image, application 1
